# Risk factors of post renal transplant anaemia among Sudanese patients, a study in three renal transplant centres

**DOI:** 10.1186/1471-2369-12-37

**Published:** 2011-08-09

**Authors:** Amin SI Banaga, Mohamed EA Yousif, Khalifa Elmusharaf

**Affiliations:** 1 Department of Medicine & Nephrology, University of Medical Sciences and Technology, Consultant Physician and Head of Haemodialysis Unit, Academy Charity Teaching Hospital, Khartoum, P.O Box. 12810, Sudan; 2 Department of Nephrology, Ibn Sina Hospital, Dr. Selma Centre for Kidney Diseases, Consultant Physician and Nephrologist, Head of the Renal Transplantation Team at Ibn Sina Hospital, Khartoum, Sudan; 3 Connecting Health Research in Africa & Ireland Consortium 'ChRAIC', PhD Researcher Health System & Policy, Department of Epidemiology, Royal College of Surgeons, Ireland

**Keywords:** Sudan, Post transplant anaemia, Erythropoietin

## Abstract

**Background:**

There is a relative lack of recent information about late post kidney transplantation anaemia (PTA), especially in the developing countries; data are scarce about the prevalence and risk factors of PTA. Sudan was a leading country in Africa and Arab world in kidney transplantation. The first kidney transplantation in Sudan was in 1973.

**Methods:**

This is a cross-sectional hospital analytic study enrolling all kidney transplanted recipients following in the transplant referral clinics at Ahmed Gassim, Selma and Ibn Sina Hospitals, Khartoum/Sudan, in the period from 1/8/2010 to 1/9/2010, clinical and laboratory data were obtained from 114 patients, anaemia was defined as Hb levels of < 13 g/dl for male patients and < 12 g/dl for female patients, exclusion criteria were pregnancy, below 18 years old patients, multiple organ transplantation, and patients with less than one year from the transplantation.

**Results:**

The study showed that 39.5% of the patients were anaemic. Univariate analysis showed that late PTA is significantly associated with not using Erythropoietin (EPO) in the pre-transplant period (p = < 0.001), history of rejection (p = 0.003), longer time from transplantation (p = 0.015), and eGFR (p < 0.0001). Multivariate analysis showed that eGFR (p = < 0.001) and not use of EPO in the pre transplant period (p < 0.001) are strong predictors of PTA. The use of Angiotensin converting enzyme inhibitors/Angiotensin receptors blockers (ACEI/ARB), immunosuppressive treatments, presence or absence of co-morbidities, donor type and donor age are not significantly associated with late PTA.

**Conclusion:**

The study concluded that late PTA is common and under recognized. Risk factors for late PTA include renal dysfunction, history of rejection, longer duration of transplantation and not using EPO in the pre-transplant period. Renal dysfunction and not using EPO in the pre-transplant period are major predictors of late PTA.

## Background

Anaemia in End Stage Renal Failure (ESRF) is mainly due to loss of the endocrine function of the kidneys that lead to deficiency of erythropoietin (EPO) and development of anaemia. Many studies pointed out the prevalence of Post transplant Anaemia (PTA) in developed countries, a Japanese study concluded that the prevalence of PTA is 20% [1], a big multicenter study conducted in 72 centers in 16 European countries [Transplant European Survey on Anaemia Management (TRESAM)] concluded that the prevalence of PTA was 38.6% [2]. In a published British study, the prevalence of anaemia was 53% at 12 months from the kidney transplantation [3]. A Turkish study concluded that prevalence of PTA was 49.3% [4], in Austria PTA was present in 39.7% [5]. Among Hungarians, PTA was 33.8% [6].

Renal dysfunction is strongly associated with development of PTA [2,7-18] and considered as a major risk factor, other risk factors like rejection [9,19,20], recent infections [21-24], longer duration from transplantation [7], immunosuppressive treatments [3,25-32], use of Angiotensin converting enzyme inhibitors/Angiotensin receptors blockers (ACEI/ARB) [2,12,13,33,34], low serum albumin [4], protein energy waste syndrome [35], and old age of the kidney donor [2] are all considered as risk factors for development of PTA.

Our aim in this study is to identify the prevalence of late PTA (> 1 year post transplant) and the risk factors of late PTA among adult Sudanese kidney recipients.

## Methods

### Study population & Data collection

This study is a cross sectional hospital base analytic study. The subjects of the study are all kidney transplanted recipients following in the transplant referral clinics at Ahmed Gassim, Selma & Ibn Sina Hospitals, Khartoum/Sudan. All patients attending the transplant referral clinics between (1/8/2010 - 1/9/2010) were interviewed by questionnaire focusing on personal and clinical data exploring (time on dialysis, receiving EPO treatment during dialysis, period of transplantation, donor age, immunosuppressive regimen, The use of ACEI/ARB, history of rejection, and presence or absence of co-morbidities) with a review of their medical files. All laboratory investigations conducted at the time of the visit such as (complete blood count, peripheral blood picture, and renal functions) were reported. Estimated Glomerular Filtration rate (eGFR) was calculated using the abbreviated modification of diet in renal disease (MDRD) study formula [36]. The research was in compliance of the declaration of Helsinki and approved by ethics and research committees in the local hospitals, an informed consent was obtained from each patient participated in the study

### Haemoglobin target

Anaemia was defined as Hb levels of < 13 g/dl for male patients and < 12 g/dl for female patients this targets were selected based on the WHO guidelines & the American Society of Transplantation [37].

### Inclusion & exclusion criteria

We included all kidney transplant recipients who received a kidney transplant and attended the referral clinic at Ahmed Gassim, Selma and Ibn Sina Hospitals between 1/8 -1/9/2010. The exclusion criteria were Pregnancy, below 18 years old patients, multiple organ transplantation and patients with less than 1 year from the transplantation.

### Statistical analysis

Categorical variables between the anaemic and non anaemic patients were compared using *X*^2 ^test, continues variables between the anaemic and non anaemic patients were compared using Student T-test. Multivariate linear regression analysis used to identify the predictors of PTA. Statistical analysis conducted using SPSS 18 software package (SPSS Inc, Chicago, IL, USA) and P value < 0.05 were considered significant.

## Results

The characteristics of the study population are shown in Table 1. Our result showed that the prevalence of late PTA was 39.5% of all Sudanese kidneys transplanted patients. Prevalence of anaemia in Ibn Sina centre was 38.7%, Selma centre was 38.5% and Ahmed Gassim centre was 40%

**Table 1:** The characteristics of the study population

Recipient gender^a^	Male 78 (68.4%) Female 36 (31.6%)
Recipient age^b^	42.4 ± 12.66

Occupation^a^	Unemployed 81 (71.1%) Non professional 16 (14%) Professional 17 (14.9%)

Donor type^a^	Living related donor: 99 (86.8%) Living unrelated: 15 (13.2%)

History of acute rejection^a^	Yes 13 (11.4%) No 101 (88.6%)

Donor age (years)^b^	28.9 ± 7.6

Use of EPO pre-transplant^a^	Yes 64 (56.1%) No 50 (43.9%)

Use of Mycofenolate mofetil^a^	Yes 31 (27.2%) No 83(72.8%)

Use of Azathioprine^a^	Yes 80 (70.2%) No 34 (29.8%)

Use of Cyclosporine^a^	Yes 45 (39.5%) No 69 (60.5%)

Use of Tacrolimus^a^	Yes 65 (57%) No 49 (43%)

Use of ACEI/ARB^a^	Yes 23 (20.2%) No 91 (79.8%)

Co-morbidities	Yes 9 (7.9%) No 105 (92%)

Time on Dialysis (months)^b^	14.6 ± 13

Period of kidney transplantation (months)^b^	49 ± 42.26

Hb g/dl^b^	12.7 ± 1.96

Creatinine mg/dl^b^	1.4 ± 0.88

Urea mg/dl^b^	39.17 ± 19.5

eGFR ml/min/1.73^2 b^	81.6 ± 34.3

^a ^Number (percentage), ^b ^Mean ± SD

A univariate analysis of risk factors associated with late PTA showed that anaemia is significantly associated with not using EPO in pre-transplant period (p < 0.001), History of rejection was also associated with PTA (p = 0.003), eGFR (p < 0.0001) and long period from transplantation (p = 0.015). No significant relation was found between anaemia and age, time on dialysis, donor age, immunosuppressive treatments, presence or absence of co-morbidities, ACEI/ARB and gender (Table 2).

**Table 2:** Risk factors of post transplant anaemia

	Anaemic group N (45)	Non anaemic group N (69)	P value
Age^b^	42.9 ± 12. 9	42 ± 12.7	0.7

Gender (M/F)^a^	31/14	47/22	0.93

Use of (EPO) pre transplant^a^	12 (18.8%)	52 (81.3%)	< 0.0001

Donor type^a^ Living related	38 (38.4%)	61 (61.6%)	0.54
	
Living unrelated^a^	8 (53.3%)	7 (46.7%)	

History of rejection^a^	10 (76.9%)	3 (23.1%)	0.003

Use of Mycofenolate mofetil^a^	10 (32.3%)	21 (67.7%)	0.33

Use of Azathioprine^a^	33 (41.2%)	47 (58.8%)	0.55

Use of Cyclosporine^a^	22 (48.9%)	23(51.1)%	0.09

Use of Tacrolimus^a^	21(32.3%)	44 (67.7%)	0.07

Use of ACEI/ARB^a^	8(34.8%)	15(65.2%)	0.6

Presence of Co-Morbidities	2(22.2%)	7 (77.8)	0.27

Time on dialysis (months)^b^	12.9 ± 11.6	15.7 ± 13.8	0.26

Time since transplantation (months)^b^	62.1 ± 51.5	40.5 ± 33.02	0.015

Donor age (years)^b^	29.3 ± 7	28.5 ± 8	0.6

eGFR (ml/min/1.73^2^	56.8 ± 20.7	97.7 ± 31.8	< 0.0001

^a ^Number (percentage), ^b ^Mean ± SD

Multivariate analysis conducted by linear regression analysis for risk factors of late PTA, using haemoglobin as dependant variable, in the linear regression model (*r = 0.626, p = < 0.0001*) eGFR (p < 0.0001) and not using EPO in the pre-transplant period (p < 0.0001) significantly predict anaemia among kidney recipients (Table 3)

**Table 3:** Liner regression model of serum haemoglobin as dependant variable (***r = 0.626, p = < 0.0001***)

Variable	β	*t*	*P value*
Age	-0.008	-0.097	0.923

Gender	0.058	0.731	0.466

eGFR	0.381	4.563	< 0.0001

Presence or absence of Co-morbidities	-0.064	-0.822	0.431

Time since transplantation	-0.031	- 0385	0.701

History of rejection	-0.123	-1.608	0.111

Use of EPO	0.376	4.736	< 0.0001

## Discussion

### Prevalence of Late PTA

Published literature on PTA is limited, especially in developing countries, this is due to lack of transplantation facilities which lead to deficiency on PTA published data, this study is the first Sudanese study on prevalence and risk factors of late PTA. Our result showed that 39.5% of kidney transplanted patients have anaemia. This high prevalence indicates the severity of this problem among Sudanese renal transplanted population; our result is higher than many of the published literature in the developed countries. Prevalence of PTA in Japan is 20% [1]. In other American study it was 26% in 5 years post transplant [7]. In the European TRESAM study it was 38% [2]. In Austria, PTA was present in 39.7% [5]. Among Hungarians, PTA was 33.8% [6]. In other hand data published from Asia and South America showed higher prevalence of PTA, in Turkey PTA was present among 49.3% of transplanted patients [4], PTA in Argentina was 42.2% [38].

### The characteristics of the study population

In our study, we reviewed 114 patients, 68% were males, Live related donors constitute 86.6% of the donor type with no cadaveric donors in our study population; this is very different from the other major published literature from the developed countries where cadaveric donors are the major donor type in kidney transplantation. 43.9% of this study population didn't receive EPO during dialysis because of financial reasons which reflects poor management of anaemia among our patients in the pre-transplant period. The mean age in this study was 42 years. Only 9 patients have co-morbidities mainly (Liver diseases, Diabetes mellitus and Heart diseases). Despite that renal transplantation improves the quality of life of the patients; unemployment rate is high among kidney recipients 71.1%

### Risk factors for PTA

After univariate analysis of the variables, history of rejection was associated with anaemia (p = 0.003), this result agrees with many of published articles which stated that rejection is a leading cause for anaemia [9,19,20].

Renal dysfunction was strongly associated with anaemia; in our study, anaemic patients have mean of eGFR of 56 ml/min/1.73^2 ^(p = < 0.0001), this result is going with the majority of published literature on PTA suggesting renal dysfunction as major cause of PTA [2,7-18].

Patients with longer duration of transplantation have more tendency to develop late PTA (p = 0.015), this is agrees with many literature which pointed out the increase prevalence of anaemia in the late post-transplant period in comparison with early post-transplant period [7], this is also can be explained by the fact that chronic allograft nephropathy can occur after long time from transplantation and that may lead to development of PTA.

In this study not using of EPO before transplantation was a major risk factor for PTA (p < 0.0001), so patients may still have significant anaemia at time of transplantation. Use of EPO pre transplant is associated with better 5 year graft survival and reduce frequency of rejection [39], concerning the level of EPO in the post transplant period, in a study compared two groups (anaemic & non-anaemic) of renal transplant recipients with normal renal functions, EPO deficiency and resistance play a causative role for PTA [40], however in other study no relation was found between administration of the EPO and anaemia, the same result favoring use of EPO only on high risk patients [41].

Multivariate analysis showed that renal dysfunction and not using EPO before transplantation were major predictors for PTA.

In this result, immunosuppressive treatment was not associated with anaemia this is contradicting with the majority of the literature which stating the strong relationship [3,25-32], however another study found no relation between anaemia and some combinations of immunosuppressive treatments [2] and perhaps live related transplantation needing less immunosuppressive treatments may be the curl print point.

In this study there was no relation between ACEI/ARB and late PTA, the literature concerning that is controversial with some favoring the link between PTA and ACEI/ARB [2,12,13,33,34] and other articles denied the relationship [15,17].

Our study had many limitations that need to be recognized, we didn't have data on iron stores and serum albumin this is mainly due to lack of financial recourses, medical data about the exact nature and details of co-morbidities was difficult to obtain, so we just asked our patients about nature and presence or absence of co morbidities. Data about numbers and treatment of rejection and history of blood transfusions was not collected.

## Conclusion

The study concluded that late PTA is common and under recognized. Risk factors for late PTA include renal dysfunction, history of rejection, longer duration of transplantation and not using EPO in the pre-transplant period. Renal dysfunction and not using EPO in the pre-transplant period are major predictors of PTA.

## Competing interests

The authors declare that they have no competing interests.

## Authors' contributions

ASIB designed the questionnaire, collected the sample, carried out the study, analyzed the data, and drafted the manuscript. MEAY directed the study, drafted and revised the manuscript. KE revised the methodology, statically analyzed the data and revised the manuscript. All authors read and approved the final manuscript

## Pre-publication history

The pre-publication history for this paper can be accessed here:

http://www.biomedcentral.com/1471-2369/12/37/prepub
